# Comparison of Oleocanthal-Low EVOO and Oleocanthal against Amyloid-β and Related Pathology in a Mouse Model of Alzheimer’s Disease

**DOI:** 10.3390/molecules28031249

**Published:** 2023-01-27

**Authors:** Ihab M. Abdallah, Kamal M. Al-Shami, Amer E. Alkhalifa, Nour F. Al-Ghraiybah, Claudia Guillaume, Amal Kaddoumi

**Affiliations:** 1 Department of Drug Discovery and Development, Harrison College of Pharmacy, Auburn University, 720 S Donahue Dr., Auburn, AL 36849, USA; 2 Modern Olives, 151 Broderick Road, Lara, VIC 3212, Australia

**Keywords:** olive oil, EVOO, oleocanthal, amyloid β, neuroinflammation, NLRP3 inflammasome, RAGE, NF-κB pathway, Alzheimer’s disease

## Abstract

Alzheimer’s disease (AD) is characterized by several pathological hallmarks, including the deposition of amyloid-β (Aβ) plaques, neurofibrillary tangles, blood–brain barrier (BBB) dysfunction, and neuroinflammation. Growing evidence support the neuroprotective effects of extra-virgin olive oil (EVOO) and oleocanthal (OC). In this work, we aimed to evaluate and compare the beneficial effects of equivalent doses of OC-low EVOO (0.5 mg total phenolic content/kg) and OC (0.5 mg OC/kg) on Aβ and related pathology and to assess their effect on neuroinflammation in a 5xFAD mouse model with advanced pathology. Homozygous 5xFAD mice were fed with refined olive oil (ROO), OC-low EVOO, or OC for 3 months starting at the age of 3 months. Our findings demonstrated that a low dose of 0.5 mg/kg EVOO-phenols and OC reduced brain Aβ levels and neuroinflammation by suppressing the nuclear factor-κB (NF-κB) pathway and reducing the activation of NOD-, LRR- and pyrin domain-containing protein 3 (NLRP3) inflammasomes. On the other hand, only OC suppressed the receptor for advanced glycation endproducts/high-mobility group box 1 (RAGE/HMGB1) pathway. In conclusion, our results indicated that while OC-low EVOO demonstrated a beneficial effect against Aβ-related pathology in 5xFAD mice, EVOO rich with OC could provide a higher anti-inflammatory effect by targeting multiple mechanisms. Collectively, diet supplementation with EVOO or OC could prevent, halt progression, and treat AD.

## 1. Introduction

Alzheimer’s disease (AD) is a progressive neurodegenerative disorder and is the most common type of dementia [1]. Approximately 10% of individuals over 65 years have AD, and its incidence continues to increase with age [2]. Clinical symptoms of AD include progressive memory deficits, cognitive dysfunction, and motor abnormalities, which could ultimately affect executive function, speech, and visuospatial orientation [2,3]. AD is characterized by two key pathological hallmarks, namely, the extracellular deposition of amyloid-β (Aβ) plaques and neurofibrillary tangles (NFTs) [4,5,6,7]. Aβ pathology results from the cleavage of the amyloid precursor protein (APP); APP is a membrane-bound protein that is processed to produce Aβ peptides such as Aβ_40_ and Aβ_42_ [8]. Produced Aβ monomers aggregate to form intermediate structures called Aβ oligomers, which eventually aggregate to form Aβ fibrils and plaques, thus contributing to AD pathology [9].

While Aβ deposition and the intraneuronal deposits of NFTs are the key players in the pathological characteristics of AD [10], they alone cannot elucidate AD’s pathogenesis, suggesting the involvement of additional pathways involved in AD pathological process [11]. Accumulating evidence supports neuroinflammation’s role in the progression of the neuropathological changes observed in AD [12,13,14]. In the initial stages of AD, glial cells positively affect Aβ elimination by phagocytosis [15]. However, upon chronic exposure to increased Aβ levels, both astrocytes and microglia become reactive, hence, activating several inflammatory pathways such as the NOD-, LRR- and pyrin domain-containing protein 3 (NLRP3) inflammasomes and receptor for advanced glycation endproducts/high mobility group box 1 (RAGE/HMGB1) pathways [16,17]. The continuous activation of astrocytes and microglia leads to changes in their phenotypes and morphology accompanied by elevated secretion of proinflammatory cytokines such as interleukin-6 (IL-6) and interleukin-1β (IL-1β) [18,19].

Moreover, the accumulation of Aβ and neuroinflammation leads to blood–brain barrier (BBB) dysfunction [20]. BBB consists of several cellular components, including endothelial cells, astrocytes, pericytes, and basement membranes [21]. The endothelial cells of the BBB are tightly connected through tight and adherence junction proteins. This tight connection restricts solute movement between the blood and the brain [21]. A key route for Aβ clearance from the brain is through its transport across the BBB, mediated by Aβ key transport proteins P-glycoprotein (P-gp) and the low-density lipoprotein receptor-related protein-1 (LRP1) [22,23,24]. Both tight and adherence junctions and transport proteins are downregulated in AD [21,22,23,24].

Several studies demonstrated the beneficial effect of the Mediterranean diet in halting and slowing AD progression [25,26]. One integral component of the Mediterranean diet that has been evaluated for its health-promoting impact is extra-virgin olive oil (EVOO). EVOO has been studied extensively for its promising health benefits. Several studies have revealed that EVOO slows memory impairment progression and improves cognitive performance in humans and AD mouse models [27,28,29,30,31,32,33]. Moreover, our studies have shown that the addition of EVOO to the diet of AD mouse models enhanced the BBB function, increased Aβ clearance, reduced its production, and reduced neuroinflammation [29,30,31]. EVOO is composed of glycerol (∼95%) and nonglycerol (∼5%) and is obtained from the first pressing of the olive fruit by mechanical means [34]. EVOO contains more than 35 phenolic compounds possessing many antioxidant and anti-inflammatory characteristics [35,36,37]. Among the phenolic compounds that were isolated and characterized is oleocanthal (OC) [37]. Oleocanthal proved effective against Aβ and related pathology in AD mouse models [28,29,30,31,38]. At 5 and 10 mg/kg doses, OC reduced brain Aβ levels, improved BBB function, and reduced neuroinflammation [28,38]. In separate studies, we evaluated the effect of OC-low and OC-rich EVOO on Aβ and related pathology in AD mouse models; both olive oils demonstrated neuroprotective effects [30,31,38].

Besides OC, EVOO contains several phenolic compounds such as hydroxytyrosol, tyrosol, oleuropein, and oleacein, to list a few, which also demonstrated neuroprotective effects when tested in vitro and in vivo [39,40,41,42,43,44,45,46,47,48]. Different grades of olive oil are available based on the phenolic content, including phenolic-free refined olive oil (ROO), phenolic-rich EVOO (but low in OC), and OC-rich EVOO. Yet, a direct comparison between these olive oils for their neuroprotective effect has not been evaluated, which is essential to clarify the impact of EVOO with different phenolics content on AD pathology. Thus, in this work, we aimed to assess and compare the effect of ROO, OC-low EVOO- and OC-enriched diet added at a low dose of 0.5 mg/kg body weight per day for three months on brain Aβ levels and neuroinflammation in a homozygous 5xFAD mouse model of AD.

## 2. Results

### 2.1. EVOO and OC Treatments Reduced Aβ Burden in 5xFAD Mouse Brains

**Figure 1:**
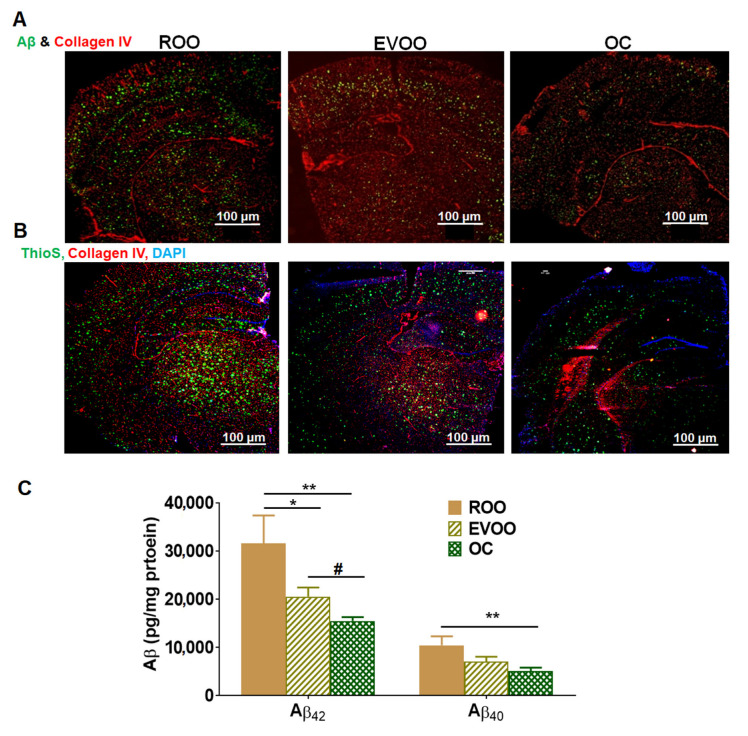
Effect of EVOO and OC consumption (0.5 µg/kg) for three months on Aβ burden in 5xFAD mouse brains. (**A**) Representative brain sections stained with 6E10 antibody to detect total Aβ load and anti-collagen IV antibody to detect microvessels. (**B**) Representative brain sections stained with ThioS to detect Aβ plaques, anti-collagen IV antibody to detect microvessels, and DAPI (blue). Scale bar, 100 µm. (**C**) Brain levels of both soluble human Aβ_40_ and Aβ_42_ levels were determined by ELISA. Data represented as mean ± SEM (n = 8 mice per group). * *p* < 0.05, ** *p* < 0.01 compared to ROO; # *p* < 0.05 compared to EVOO.

Homozygous 5xFAD mice were fed with refined olive oil (ROO), OC-low EVOO (0.5 mg total phenolic content/kg; hereafter EVOO), and OC (0.5 mg OC/kg) for 3 months starting at the age of 3 months. Compared to ROO-treated mice, EVOO and OC treatments reduced total Aβ load in 5xFAD mouse brains as determined by immunofluorescence analysis. As shown in Figure 1A, EVOO and OC treatments reduced total Aβ (detected by 6E10 antibody) levels in brain sections. In addition, both EVOO and OC reduced Aβ plaques as determined by Thioflavin S (ThioS) staining (Figure 1B). Further analysis was performed to evaluate the effect of treatments on the levels of soluble Aβ_40_ and Aβ_42_ by ELISA (Figure 1C). Compared to the ROO group, EVOO significantly reduced Aβ_42_ by 35%; however, it didn’t reach significance for its effect on Aβ_40_ levels, which decreased by 30% due to the high variability in the ROO group; OC, on the other hand, was able to reduce both isoforms significantly by about 50%. While no significant difference was observed between EVOO and OC on reduced Aβ_40_ levels, OC demonstrated a significant reduction in Aβ_42_ levels compared to EVOO.

### 2.2. EVOO and OC Enhanced BBB Function in 5xFAD Mice Brains

**Figure 2:**
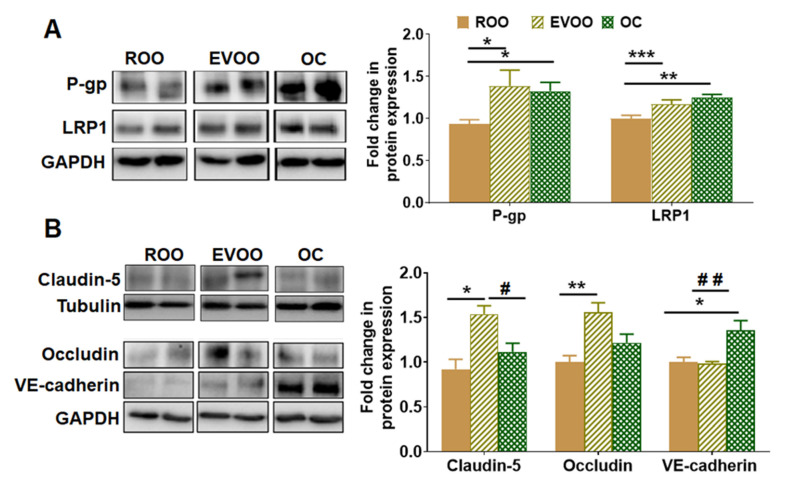
Effect of EVOO and OC consumption (0.5 µg/kg) for three months on Aβ clearance, and tight and adherence junction proteins in 5xFAD mouse brains. (**A**) Representative blots and densitometry analysis of Aβ major transport proteins across the BBB, P-gp, and LRP1. (**B**) Representative blots and densitometry analysis of the tight junction proteins claudin-5 and occludin and the adherence protein VE-cadherin (n = 6–8 mice per group). Values were normalized to the ROO group (1.0). Data are presented as mean ± SEM. * *p* < 0.05, ** *p* < 0.01, *** *p* < 0.001 compared to ROO; and # *p* < 0.05, ## *p* < 0.01 compared to EVOO.

To monitor and compare the effect of EVOO and OC on the BBB function, we evaluated their effect on the expression of Aβ major transport proteins P-gp and LRP1, the tight junction proteins claudin-5 and occludin, and the adherence junction protein VE-cadherin by Western blot. As shown in Figure 2A, EVOO and OC increased the expression of P-gp by 33%–39% compared with ROO-treated mice. For their effect on LRP1, while the effect was mild, both EVOO and OC significantly increased LRP1 levels by 17% and 25%, respectively. 

Regarding their effect on tight and adherence junction proteins, EVOO significantly increased claudin-5 and occludin expressions by 44% and 56%, respectively, while OC increased VE-cadherin significantly by 35% (Figure 2B).

### 2.3. EVOO and OC Reduced the Amyloidogenic and Enhanced the Non-Amyloidogenic Pathways in 5xFAD Mouse Brains

**Figure 3:**
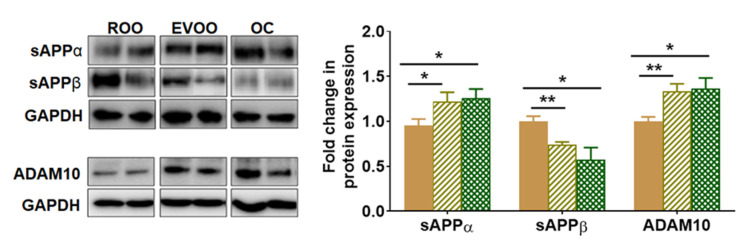
Effect of EVOO and OC consumption (0.5 µg/kg) for three months on Aβ production proteins in 5xFAD mouse brains. Representative blots and densitometry analysis of sAPPα, sAPPβ, and ADAM10 (n = 6 mice per group). Values were normalized to the ROO group (1.0). Data are presented as mean ± SEM. * *p* < 0.05, ** *p* < 0.01 compared to ROO.

The effect of EVOO and OC on Aβ production was evaluated by monitoring changes in the expression of sAPPα, sAPPβ, and ADAM10 (α-secretase) by Western blot. APP processing undergoes enzymatic cleavages by ADAM10 to produce sAPPα (the non-amyloidogenic pathway) and by β-secretase to produce sAPPβ followed by γ-secretase cleavage to form Aβ peptides (the amyloidogenic pathway). As shown in Figure 3, the consumption of EVOO- and OC-enriched diet significantly increased the expression of ADAM10 by approximately 35% compared with the ROO group. This increase was associated with a significant increase in sAPPα by 25% in mice that consumed EVOO and OC brains. In addition, EVOO and OC significantly reduced sAPPβ levels by 30%–40% (Figure 3). There was no significant difference between EVOO and OC in their effect on Aβ production.

### 2.4. EVOO and OC Increased the Expression of Synaptic Markers in 5xFAD Mouse Brains

**Figure 4:**
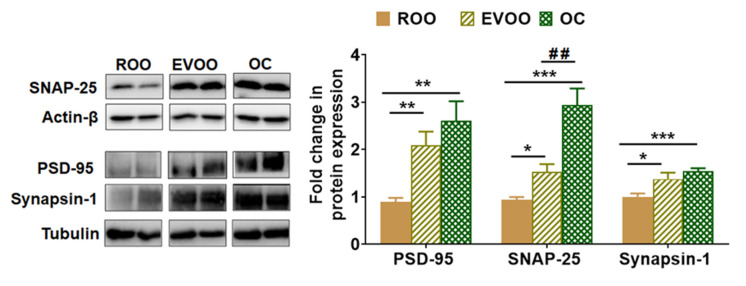
Effect of EVOO and OC consumption (0.5 µg/kg) for three months on synaptic markers in 5xFAD mouse brains. Representative blots and densitometry analysis of PSD-95, SNAP-25 and synapsin-1 (n = 6 mice per group). Values were normalized to the ROO group (1.0). Data are presented as mean ± SEM. * *p* < 0.05, ** *p* < 0.01, *** *p* < 0.001 compared to ROO; and *## p* < 0.01 compared to EVOO.

Two pre-synaptic markers (SNAP-25 and synapsin-1) and one post-synaptic marker (PSD-95) were evaluated. As shown in Figure 4, Western blot findings demonstrated EVOO, and OC significantly increased the expression of the neurosynaptic markers PSD-95 by 2- and 2.5-fold, SNAP-25 by 1.5- and 2.9-fold, and synapsin-1 by 1.3 and 1.4-fold, respectively, when compared with the ROO enriched-diet. While both EVOO and OC induced the synaptic markers, OC demonstrated a significant increase in SNAP-25 expression compared with EVOO. 

### 2.5. EVOO and OC Reduced Neuroinflammation in 5xFAD Mouse Brains

**Figure 5:**
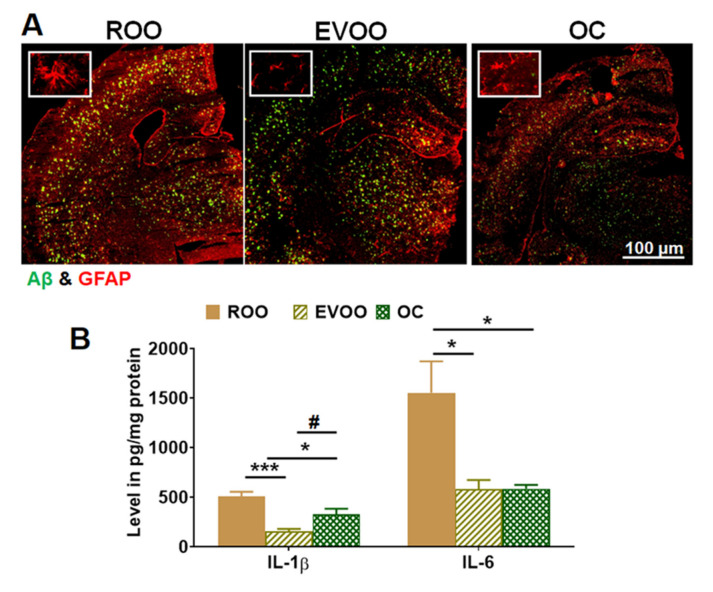
Effect of EVOO and OC consumption (0.5 µg/kg) for three months on astrocytes activation and cytokines in 5xFAD mouse brains. (**A**) Representative brain sections stained with anti-GFAP antibody and anti-Aβ (6E10 antibody) to detect activated astrocytes (seen at higher magnification in the closed inserts, 500 μm). (**B**) Brain levels of the cytokines IL-1β and IL-6 as determined by ELISA (n = 6 mice per group). Values were normalized to the ROO group (1.0). Data are presented as mean ± SEM. * *p* < 0.05, *** *p* < 0.001 compared to ROO; and *# p* < 0.05 compared to EVOO.

Astrocyte activation is recognized by increased glial fibrillary acidic protein (GFAP) with an elongated shape and thick branches. As shown in Figure 5A, in comparison to the ROO group, EVOO and OC significantly reduced astrocyte activation and ameliorated the astrocyte shape in mouse brains. Besides astrocytes activation, stimuli such as Aβ and the proinflammatory cytokines IL-1β and IL-6 activate NLRP3, which promotes the inflammasome complex formation. Thus, in this work, we evaluated and compared the effect of EVOO and OC on the production of proinflammatory cytokines by ELISA, and NLRP3 by Western blotting. As shown in Figure 5B, mice treated with EVOO and OC significantly reduced IL-1β levels by approximately 70% and 40%, respectively, and reduced IL-6 levels by about 60% (Figure 5B). The results demonstrated that the effect of EVOO on reducing IL-1β is significantly greater than OC. 

**Figure 6:**
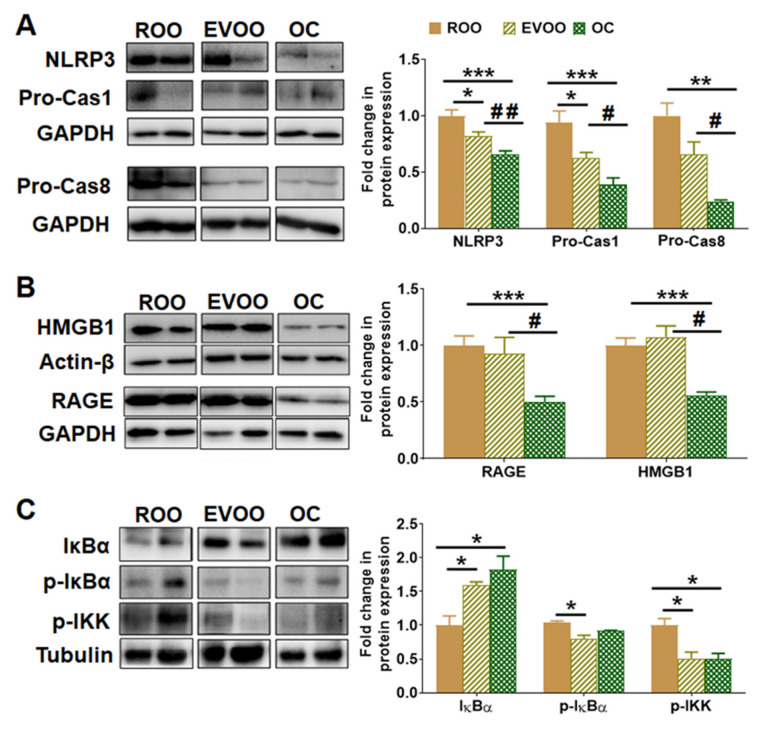
Effect of EVOO and OC consumption (0.5 µg/kg) for three months on the neuroinflammation markers NLRP3, RAGE, and HMGB1, and NF-κB pathway in 5xFAD mouse brains. (**A**) Representative blots and densitometry analysis of NLRP3, pro-caspase 1 (Pro-Cas1), and pro-caspase 8 (Pro-Cas8) in brain homogenates. (**B**) Representative blots and densitometry analysis of RAGE and HMGB1, and (**C**) Representative blots and densitometry analysis of IκBα, p-IκBα, and p-IKKα (n = 6–8 mice per group). Values were normalized to the ROO group (1.0). Data are presented as mean ± SEM. * *p* < 0.05, ** *p* < 0.01, *** *p* < 0.001 compared to ROO; and # *p* < 0.05, ## *p* < 0.01 compared to EVOO.

For their effect on NLRP3 inflammasome activation, OC demonstrated a significant reduction in NLRP3, pro-caspase 1, and pro-caspase 8 when compared to ROO and EVOO. As shown in Figure 6A, OC significantly reduced NLRP3 levels by 40% compared with the ROO group, while EVOO showed a 15% reduction. Reduced NLRP3 levels were associated with a significant decrease in pro-caspase 1 levels by 40% and 67% and pro-caspase 8 by 42% and 79% by EVOO and OC-treated mice, respectively.

### 2.6. OC Reduced RAGE and HMGB1 Expressions in 5xFAD Mouse Brains

Ligands, including Aβ and high-mobility group box protein 1 (HMGB1), interact with the receptor for AGEs (RAGE) and directly induce inflammation. Thus, we next examined EVOO and OC effects on the expression of RAGE and HMGB1 in the brain homogenates of the 5xFAD mice. As shown in Figure 6B, RAGE and HMGB1 expressions were significantly reduced by approximately 40%–50% by OC; however, EVOO did not alter the levels of either protein compared to the ROO group. These results suggest that besides suppressing NLRP3 inflammasomes, OC suppresses the RAGE/HMGB1 pathway. 

### 2.7. EVOO- and OC-Reduced Neuroinflammation Is Mediated by NF-κB Pathway in 5xFAD Mouse Brains

To determine whether the observed effect is mediated through attenuating NF-κB pathway, we evaluated the impact of the treatments on three major proteins from the NF-κB pathway, namely IκBα, p-IκBα (Ser32), and p-IKKβ. As shown in Figure 6C, EVOO and OC significantly increased the expression of total IκBα by approximately 60% and 80%, respectively, and both reduced the expression of p-IKKβ by 50% compared with the ROO group. This significant reduction in p-IKKβ was associated with a significant decrease in p-IκBα by approximately 20% in the EVOO group; however, the effect of OC did not reach a significant level. The results also demonstrated no significant difference between EVOO and OC on the NF-κB pathway, which suggests that EVOO and OC effect could be mediated, at least in part, by suppressing the NF-κB pathway. 

## 3. Discussion

AD is a complex neurodegenerative disorder that could be affected by modifiable and non-modifiable risk factors, thus influencing the disease susceptibility [49]. AD has several hallmarks, including Aβ plaques, NFT, compromised BBB, widespread activated glial cells, synaptic dysfunction, neuroinflammation, and neuronal death [4,5,6,7,10,14]. 

The Mediterranean diet has several beneficial effects and has been associated with a slower rate of cognitive impairment and dementia [50,51]. Olive oil is the primary fat source in the Mediterranean diet, which possesses anti-inflammatory, antioxidant, and neuroprotective effects [25,26,27,52]. Several studies by us and others have reported the beneficial effects of EVOO and its phenolic components, especially OC, in AD mouse models [28,29,30,31,38,53,54]. Previous findings from separate studies demonstrated that treating AD mouse models with OC-rich EVOO or high doses of OC (in saline as a vehicle) reduced Aβ and related pathology [28,30,31,38]. In addition, a few studies have tested the effect of EVOO in subjects with mild cognitive impairment (MCI) and reported the beneficial effect of EVOO containing variable phenolic content. EVOO consumption modulated plasma Aβ and hyperphosphorylated tau levels, enhanced the BBB function, reduced blood inflammatory and oxidative stress biomarkers, and improved cognitive function in MCI [32,33,55]. However, a direct comparison between OC-low EVOO and OC has not been assessed. Thus, in this study, we aimed to evaluate and compare the effect of equivalent doses of OC (spiked in ROO as the vehicle) and EVOO phenolic content present in OC-low EVOO on brain Aβ levels and neuroinflammation in homozygous 5xFAD mice characterized with aggressive Aβ pathology at an early age. Our findings from this work demonstrated the following: (a) feeding mice with OC-low EVOO- or OC-enriched diet reduced brain Aβ levels, astrocytes activation, and neuroinflammation, (b) at the administered low doses, both EVOO (0.5 mg phenolic content/kg) and OC (0.5 mg/kg) demonstrated comparable effects, (c) the anti-inflammatory effect of EVOO and OC is mediated, at least in part, by suppressing NF-κB pathway and NLRP3 inflammasome activation; however, only OC suppressed RAGE/HMGB1 pathway, which infers that OC could possess a greater effect against neuroinflammation compared with EVOO. 

Several studies reported that BBB disruption plays a pivotal role in AD pathology [56,57] and suggested that its breakdown is an early event in advanced-aged human brains and contributes to cognitive decline [58]. Findings from our study indicate that EVOO- and OC-enriched diets increased BBB’s tightness through significant upregulation of tight and adherence junction proteins. At the doses administered, while EVOO induced the expression of claudin-5 and occludin, OC induced VE-cadherin. Compared with our previous findings with OC-rich EVOO, which demonstrated a significant increase in claudin-5 expression [30,31], the current data suggest that the induced reported increase in claudin-5 could be mediated by EVOO-phenols, and not OC. 

Increased Aβ levels in the brains of AD patients could be initiated by the imbalance between Aβ production and its clearance, which subsequently could lead to brain Aβ accumulation [59,60]. Here, we examined the effect of OC-low EVOO and OC on brain Aβ, proteins involved in its clearance across the BBB, and proteins related to its production. While the obtained results are consistent with our previous reports, they also suggested a lack of difference between EVOO and OC for their effect on Aβ production and clearance across the BBB. EVOO and OC reduced total Aβ, Aβ deposits, and soluble Aβ_40_ and Aβ_42_. Such a decrease in Aβ was associated with increased expression of P-gp and LRP1, implying improved BBB function. In addition, EVOO and OC reduced the production of Aβ by shifting the processing of APP toward the non-amyloidogenic pathway. This effect was associated with a significant reduction in sAPPβ. These findings are consistent with our previous studies reporting that OC-rich EVOO significantly elevated sAPPα and ADAM10 in the AD mouse model TgSwDI [30]. 

There is tremendous evidence that neuroinflammation is a crucial factor in AD pathology. Under normal physiological conditions, glial cells have a phagocytic function [61]. However, in AD brains, glial cells become activated and secrete several proinflammatory cytokines, such as IL-6, IL-1β, and TNF-α, and other oxidative stress markers, eventually leading to neuronal death [61]. EVOO and OC reduced astrocyte activation as demonstrated by GFAP and cytokines levels. To clarify the affected neuroinflammatory pathway(s) that played a role in the observed effect, at least in part, we assessed NLRP3 inflammasome and RAGE/HMGB1 pathways, both of which are mediated by NF-κB pathway activation. Advance glycated end-products (AGEs) up-regulation has been associated with aging and neurodegenerative diseases, including AD [62,63,64]. In AD, the level of AGEs is significantly higher when compared with normal brains [65,66]. In addition, RAGE is highly expressed in activated astrocytes. RAGE is also expressed in different brain cells, including endothelial cells of the BBB, neurons, and microglia [65]. Ligands such as Aβ and HMGB1 proteins have been shown to interact and increase the expression and activity of RAGE [67,68,69,70]. HMGB1 release from various cells, including astrocytes and microglia, contributes to AD via its binding to RAGE, which activates inflammatory responses [71,72]. Upon interactions, Aβ and HMGB1 activate the NF-κB pathway [73,74,75]. RAGE induced-activation of NF-κB promotes the expression of proinflammatory cytokines, which induces a prolonged activation and promotion of signaling mechanisms for cell damage and regulates NLRP3 inflammasome activation [76,77,78]. Furthermore, HMGB1 and Aβ could directly induce NLRP3 activation and promote the formation of the inflammasome complex, which results in the activation of caspase 1 and caspase 8 and the production of proinflammatory cytokines IL-1β, IL-6, and IL-18 [76,79]. The activation of NLRP3 inflammasomes enhances AD progression by mediating chronic inflammatory responses [77], which are partly involved in restricting glial function, and mediating synaptic dysfunction and cognitive impairment [77], as well as BBB dysfunction [80]. Therefore, blocking RAGE and NLRP3 inflammasome activation could effectively interfere with the progression of AD. Our study findings demonstrated that only OC suppressed RAGE/HMGB1 pathway, while OC-low EVOO did not alter this pathway. In addition, the effect of OC on NLRP3 inflammasome suppression was significantly greater than that of OC-low EVOO. In the brains of EVOO- and OC-treated mice, IL-1β levels were significantly decreased when compared with the ROO group. This effect was associated with a significant reduction in the expression of two major pro-caspases responsible for the increased formation of IL-1β via the activation of caspase-1 [81] and caspase-8 [82]. Reduced neuroinflammation by both EVOO and OC was associated with a comparable reduction in the NF-κB pathway.

Besides OC, EVOO contains many other phenolic compounds. Among the most studied EVOO-phenols for their effect against AD pathology are hydroxytyrosol, tyrosol, oleuropein, oleacein, and luteolin [39,40,41,42,43,44,45,46,47,48]. Findings from in vitro and in vivo studies with these phenols demonstrated their effect against Aβ and related pathology, where they were able to reduce Aβ levels by blocking Aβ aggregation, reduce Aβ production, and increase its clearance by different mechanisms, including autophagy and across the BBB. Furthermore, EVOO-phenols, including OC, reduced oxidative stress and neuroinflammation by targeting nuclear factor erythroid 2–related factor 2 (NRF2), nitric oxide (NO), Janus kinase/signal transducer and activator of transcription (JAK/STAT), and mitogen-activated protein kinase (MAPK) signaling pathways, to list a few, which could synergistically or additively alleviate AD pathology [39,40,41,42,43,44,45,46,47,48]. The phenols mentioned above exist in the EVOO examined in this study. When tested at an equivalent dose to ROO spiked with OC (0.5 mg/kg), both demonstrated comparable effects against Aβ pathology.

This study has a few limitations. First is the lack of behavioral studies to compare the effect of EVOO and OC on memory function. Other limitations include the lack of a control group that did not receive any olive oil and the use of male-only mice in the study. Indeed, additional studies are necessary to confirm our findings and to assess and compare different olive oils with different phenolic content.

In conclusion, our findings confirm previously reported results on EVOO and OC to prevent and/or treat AD. In addition, and for the first time, our findings demonstrate that compared to ROO, at equivalent doses, OC-low EVOO and OC effectively reduced Aβ and related pathology. Furthermore, we showed that low doses of EVOO phenolics and OC could positively impact AD-related pathology. Both EVOO and OC reduced neuroinflammation by suppressing NLRP3 inflammasomes and NF-κB pathways. In comparison, OC demonstrated an additional effect by suppressing RAGE/HMGB1 pathway. Collectively, our current and previous findings implicate that diet supplementation with EVOO low (but containing other phenols) or rich in OC could have a beneficial effect against AD.

## 4. Materials and Methods

### 4.1. Materials

Western blotting reagents, including buffers and gels, were obtained from Bio-Rad Laboratories (Hercules, CA, USA). NP-40 lysis buffer and 4x Laemmle sample buffer were purchased from Alfa Aesar (Haverhill, MA, USA). ThioS, bovine serum albumin (BSA), and donkey serum were purchased from Sigma-Aldrich (St. Louis, MO, USA). Non-fat dry milk was purchased from Santa Cruz Biotechnology (Dallas, TX, USA). Protease inhibitor and FemtoLUCENT™ PLUS-HRP were obtained from G-Biosciences (St. Louis, MO, USA). Pierce ECL WB substrate was purchased from Thermo-Fisher Scientific (Waltham, MA, USA). All other chemicals were purchased from VWR (Radnor, PA, USA) or Fisher Scientific (Hampton, NH, USA). Antibodies used for Western blot (WB) and IHC are summarized in Table 1.

### 4.2. Animals

All animal experiments and procedures were approved by the Institutional Animal Care and Use Committee of Auburn University and according to the National Institutes of Health guidelines. The mouse model used for this study is the homozygous 5xFAD mouse. The heterozygous 5xFAD mouse model rapidly develops severe Aβ-related pathology and accumulates high levels of Aβ, beginning around 1.5 months of age [83]. Astrogliosis and microgliosis develop parallel with Aβ plaque deposition at approximately two months of age. 5xFAD mice develop an APP gene dosage-dependent aggravation of the neurological phenotype. Compared to the heterozygous mice, homozygous 5xFAD mice develop amyloid pathology much more rapidly with an aggravated neuropathological and behavioral phenotype [84]. The homozygous 5xFAD mice were produced by breeding heterozygous 5xFAD (Jackson Laboratory; Ban Harbor, ME), and the offspring were confirmed by genotyping. The mice were housed in plastic containers under standard conditions, 12-h light/dark cycle, 22 °C, and 35% relative humidity, with ad libitum access to water and food. For the experiments, mice received EVOO- and OC-enriched diet for 3 months starting at the age of 3 months.

Male 5xFAD mice were divided into three groups (n = 10 mice/group). Group 1 received ROO (containing <10 mg/kg total biophenols; vehicle group) mixed with a regular powdered diet (Teklad Laboratory diets, Harlan Laboratories, Madison, WI, USA); group 2 received EVOO mixed with the powdered food; and group 3 received OC spiked in the refined olive oil that was then mixed with the powdered food. The phenolic compounds present in the EVOO and their concentrations are listed in Table 2. For the studies, EVOO that is low in OC was used with total phenolic content of 540 mg/kg, with OC presenting less than 35 mg/kg. For group 3 treatment, OC was spiked at 540 mg/kg in olive oil to produce a concentration equivalent to the total phenols in the EVOO used in this study. The olive oils were obtained from Boundary Bend Olive Pty (Australia). ROO-, EVOO- and OC-enriched diets were prepared based on the dietary intake of olive oil in the Greek population, that is 50 g/day [85], which resulted in a daily dose of 0.5 mg/kg body weight of total phenols (in EVOO) and OC. Mice were fed with ROO-, EVOO- and OC-enriched diet beginning at three months and continued for three months to end the treatment at six months. The enriched diet was changed every day to maintain freshness.

### 4.3. Western Blot Analysis

Brain tissues were collected and homogenized, as reported previously [46]. Briefly, brain tissues were lysed with NP-40 lysis buffer containing 1x protease inhibitors followed by centrifugation at 20,800× *g* for 20 min at 4 °C. The supernatant was collected and stored at −80 °C until use for analysis. Pierce BCA Protein Assay kit was used to quantify the total protein content. 

For Western blotting, 30 μg of sample protein were loaded and resolved on 10% SDS-polyacrylamide gel, then transferred electrophoretically onto PVDF membranes. Membranes were blocked using 1% milk for 1 h at room temperature; membranes were then incubated overnight at 4 °C with primary antibodies. Primary antibodies used for immunolabeling are listed in Table 1. For detection, HRP-labeled secondary antibodies were used (Table 1). The bands were visualized using the ChemiDoc MP Imaging System (Bio-Rad). The immunoreactive bands were quantified by densitometric analysis using Image Lab Software V.6.0 (Bio-Rad). The results were expressed as a fold change in protein level compared to the ROO group after normalization to the housekeeping proteins.

### 4.4. Immunofluorescence Staining and Analysis

Brain cryosections of 16 μm-thick were prepared by ThermoScientific™ HM525 NX Cryostat (Waltham, MA, USA). We used a similar staining protocol to our previous studies [46]. In brief, brain sections were fixed with methanol at −20 °C for 10 min, followed by washing with phosphate-buffered saline and blocking with 10% donkey serum for 1 h at room temperature. Primary and secondary antibodies used for these experiments are listed in Table 1. The brain sections were double stained with Alexa Fluor 488 labeled 6E10 human specific anti-Aβ antibody (1:1000 dilution) to detect total Aβ, and rabbit polyclonal collagen IV antibody (1:200) to detect brain microvessels for 2 h followed by the donkey polyclonal Alexa Flour 647 antibody to rabbit IgG (1:200). To detect Aβ plaques, brain sections were stained with a freshly prepared and filtered 0.02% ThioS solution in 70% ethanol for 30 min. Sections were washed in 70% ethanol for 15 min and covered with coverslips for imaging. To detect reactive astrocytes, sections were stained with rabbit anti-GFAP polyclonal IgG (1:100) followed by anti-rabbit IgG−CFL594 secondary antibody. Fluorescent images were captured using a Nikon Eclipse TiS inverted fluorescence microscope (Melville, NY).

### 4.5. Enzyme-Linked Immunosorbent Assay (ELISA)

Soluble Aβ_40_ and Aβ_42_ levels in brain homogenates were quantified using commercially available ELISA kits according to the manufacturer’s instructions (R&D Systems). In addition to Aβ, the proinflammatory cytokines IL-1β and IL-6 levels in mice brain homogenates were determined using ELISA according to the manufacturer’s instructions (R&D Systems). All samples were run at least in duplicate and corrected to the total protein amount in each sample using the BCA assay.

### 4.6. Statistical Analysis

Data analysis was performed using GraphPad Prism v5.0 software. The experimental results were statistically analyzed for the significant difference using Student’s t-test. All *p* values were statistically significant at *p* < 0.05. The results are presented as mean ± SEM. 

## Figures and Tables

**Table 1:** List of antibodies.

Antibody	Company
**Western blot**	
Anti-mouse IgG (H+L) secondary antibody, HRP-labeled	Invitrogen (Waltham, MA, USA)
Anti-rabbit IgG (H+L) secondary antibody, HRP-labeled	Invitrogen
ADAM10	Santa Cruz Biotechnology (Dallas, TX, USA)
Claudin-5	Invitrogen
GAPDH	Invitrogen
HMGB1	Santa Cruz Biotechnology
IκB-α	Cell Signaling (Danvers, MA, USA)
LRP1	Abcam (Waltham, MA, USA)
NLRP3	Cell Signaling
Occludin	Invitrogen
p- IκB-α	Cell Signaling
P-gp	BioLegend (San Diego, CA, USA)
p-IKK	Cell Signaling
Pro-caspase 1	Santa Cruz Biotechnology
Pro-caspase 8	Santa Cruz Biotechnology
PSD-95	Invitrogen
RAGE	Santa Cruz Biotechnology
sAPP-α	IBL America (Minneapolis, MN, USA)
sAPP-β	IBL America
SNAP-25	Invitrogen
Synapsin-1	Cell Signaling
VE-cadherin	Santa Cruz Biotechnology
**Immunofluorescence staining**	
Alexa-fluor 488-labeled 6E10	BioLegend
Anti-goat IgG−CFL594	Santa Cruz Biotechnology
Anti-goat HRP-labeled secondary	R&D Systems (Minneapolis, MN, USA)
Anti-collagen-IV	EDM-Millipore (Burlington, MA, USA)
Anti-rabbit IgG-CFL594	Santa Cruz Biotechnology
GFAP	Santa Cruz Biotechnology

**Table 2:** Phenolic compounds and their concentrations present in EVOO.

Biophenols (mg/kg)	ROO	EVOO
Hydroxytyrosol		5.1
Tyrosol		3.1
Vanillic acid and Caffeic acid		1.8
Vanillin		1.5
Para-coumaric acid		2.7
Ferulic acid		4.2
Decarboxymethyl oleuropein aglycone oxidized dialdehyde form		34.1
Oleacein		85.2
Oleuropein		29.9
Oleuropein aglycone, dialdehyde form		49.3
Tyrosol acetate		24.9
Decarboxymethyl ligstroside aglycone, oxidised dialdehyde form		37.1
Oleocanthal		33.9
Cinnamic acid		59.1
Ligstroside aglycone, dialdehyde form		12.7
Oleuropein aglycone, oxidized aldehyde, and hydroxylic form		17.4
Luteolin		36
Oleuropein aglycone, aldehyde, and hydroxylic form		58.2
Ligstroside aglycone, oxidised aldehyde, and hyroxylic form		15.1
Apigenin		16.6
Ligstoside aglycone, aldehyde and hydroxylic form		11.4
**Total biophenol content mg/kg**	**<10**	**539.3**

## Data Availability

The data presented in this study are available within the article text and figures.

## Sample Availability

ROO, EVOO and OC are commercially available.
